# Enterohemorrhagic *Escherichia coli* O157:H7 responds to norepinephrine gradients by tRNA reprogramming and codon-biased translation of virulence genes

**DOI:** 10.1128/msystems.01418-25

**Published:** 2026-04-22

**Authors:** Abigail E. McShane, Chi-Kong Chan, Ruixi Chen, Michael S. DeMott, Thomas J. Begley, Peter C. Dedon

**Affiliations:** 1 Department of Biological Engineering, Massachusetts Institute of Technology 2167, Cambridge, Massachusetts, USA; 2 The RNA Institute and Department of Biological Sciences, University at Albany 1084, Albany, New York, USA; 3 Singapore-MIT Alliance for Research and Technology Antimicrobial Resistance Interdisciplinary Research Group, Singapore, Singapore; Chinese Academy of Sciences, Shanghai, China

**Keywords:** EHEC, tRNA, norepinephrine, virulence, type III secretion, translational regulation

## Abstract

Enterohemorrhagic *Escherichia coli* O157:H7 (EHEC) is a low-infectious-dose foodborne pathogen that uses norepinephrine gradients in the gut to locate sites for colonization and activate expression of virulence factors. Here, we identified features of transfer RNA (tRNA) reprogramming and codon-biased translation that play a role in this infectious process. Multivariate statistical analysis of genome-wide codon usage patterns revealed five gene clusters with unique codon biases, with one cluster containing all Locus of Enterocyte Effacement virulence genes that are all strongly biased for A/U-ending codons. EHEC cultures were then exposed to a norepinephrine gradient to induce type III secretion, as well as chemotaxis and flagellar motility, followed by a multi-omic interrogation of changes in tRNA modifications, tRNA abundance, and the proteome. We observed altered levels of multiple tRNA wobble modifications involved in G/C- versus A/U-ending codon recognition and a corresponding shift in the proteome toward genes enriched in A/U-ending codons. Our studies suggest EHEC can alter its tRNA pool in response to cues from the host, fine-tuning translational efficiency of its virulence program.

**IMPORTANCE:**

Regulation of microbial physiology and virulence during environmental changes has typically been ascribed to transcriptional mechanisms. Using convergent multi-omic technologies, we have discovered mechanisms of translational regulation of gene expression that regulate cell phenotype. Here, we applied these technologies to define mechanisms of translational regulation in the *Escherichia coli* O157:H7 response to norepinephrine exposure known to induce virulence.

## INTRODUCTION

Establishing infection requires precise, multi-layered coordination of virulence factor expression. Enterohemorrhagic *Escherichia coli* O157:H7 (EHEC) is a particularly successful foodborne pathogen, causing diarrheal illness that can progress to devastating hemorrhagic colitis and hemolytic uremic syndrome. Unlike many other gut pathogens—and even other *E. coli* pathotypes—EHEC has an exceptionally low infectious dose, requiring as few as 10–100 colony-forming units (CFU) to establish infection (1, 2). This remarkable efficiency stems from its ability to integrate diverse environmental cues to precisely regulate expression of its immunogenic and energetically costly Type III Secretion System (T3SS), thereby promoting formation of attaching and effacing lesions on colonic epithelial cells.

Most of EHEC’s T3SS is encoded on a pathogenicity island called the Locus of Enterocyte Effacement (LEE), which is comprised of five operons that are subject to multiple levels of regulation. At the transcriptional level, the LEE is primarily controlled by the master regulators Ler and GrlA/R, but its expression is further modulated by global stress-response regulators and environmental sensing systems such as histidine kinases. As a result, LEE transcription is sensitive to host- and microbiome-derived cues, including oxygen levels, pH, temperature, nutrient availability, adrenergic hormones, and the microbial metabolite indole (3–6). In parallel, post-transcriptional regulation provides additional flexibility. EHEC encodes hundreds of small regulatory RNAs (sRNAs), which allow for rapid regulation of protein expression through diverse mechanisms (7, 8). For example, sRNA DicF promotes LEE expression under low oxygen conditions by disrupting intramolecular interactions that normally inhibit expression of PchA, a Ler transcriptional activator (9). Others include GlmY/Z, EvrZ, and MavR, and there are many more that do not directly alter LEE expression but are critical for full virulence and survival under stress (5, 8, 10).

Translational regulation of gene expression has emerged as another mode of regulation proving to be an important player in microbial physiology and virulence, with stress-induced changes in the transfer RNA (tRNA) pool regulating translation of codon-biased mRNAs encoding stress response genes (11). As adaptors linking mRNA codons to corresponding amino acids, tRNAs are heavily decorated with ribonucleoside modifications, particularly at the wobble position, with 8–10 modifications per tRNA molecule chosen from about 45–50 available in the microbial epitranscriptome (11). tRNA modifications can expand or restrict the decoding capacity of a single tRNA molecule to specific codons, with dynamic changes in tRNA modifications in response to stress or stimuli driving translation of families of response genes enriched with the cognate synonymous codons (11, 12). This is illustrated in *Mycobacterium bovis*, where hypoxia causes an increase in the wobble modification cmo^5^U in tRNA-Thr(UGU), which increases decoding capacity for the ACG codon over ACA. The latter is enriched in the *dosR* regulator of hypoxic bacteriostasis, leading to increased translation of DosR that drives transcription of a regulon enabling the bacterium to enter a state of non-replicating persistence (13). Beyond this, several studies have shown that writer enzymes that install tRNA modifications are important contributors to virulence in various bacterial pathogens including *Escherichia coli, Pseudomonas putida, Pseudomonas aeruginosa, Shigella flexneri,* and *Salmonella enterica*, but the mechanistic basis in many cases remains unclear (14–17).

**Fig 1:**
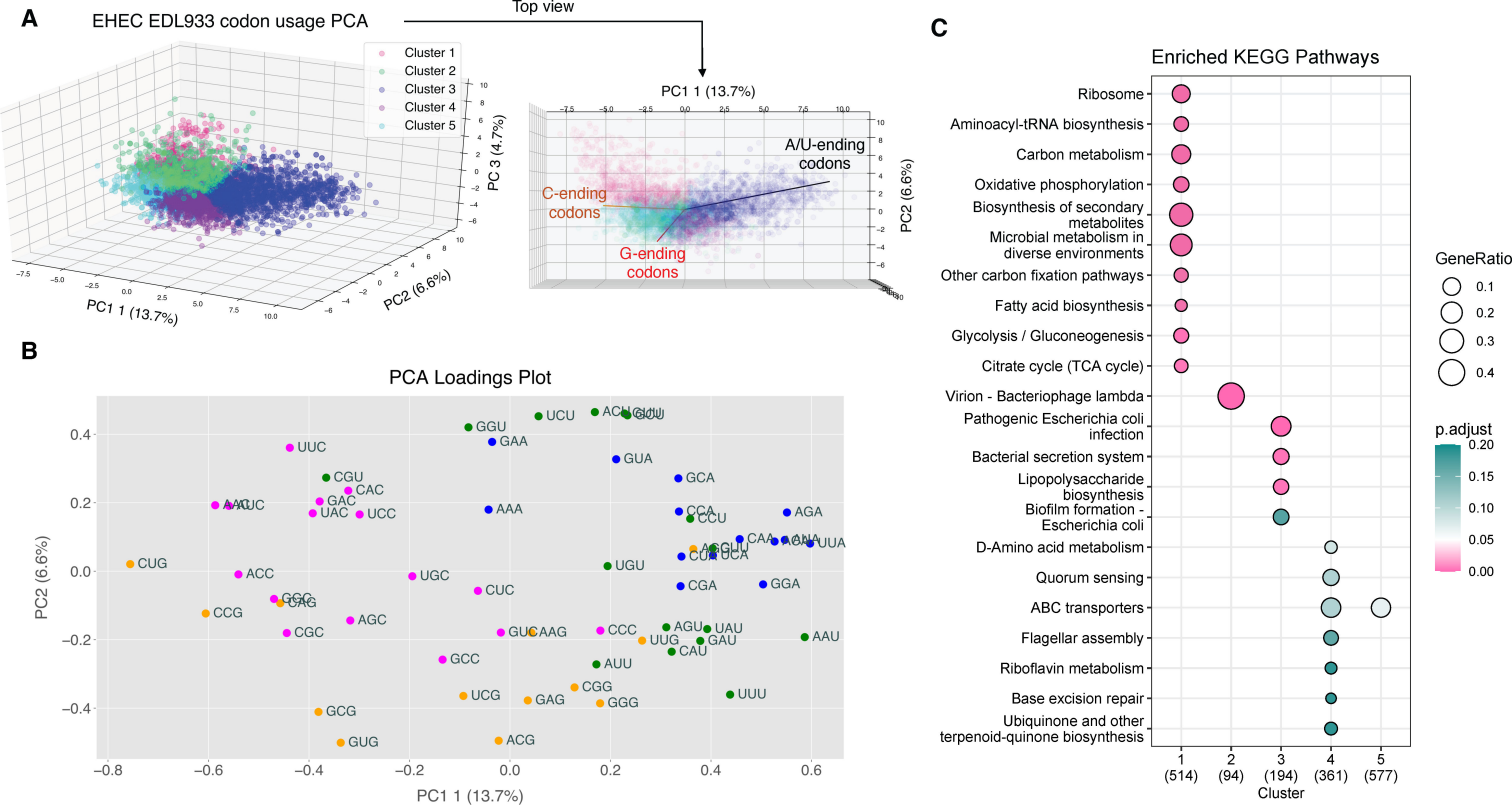
The EDL933 genome exhibits biased codon usage. (**A**) Three-component PCA scores plot of EDL933 genes based on codon *Z*-score data, colored by assigned k-means clusters from Fig. S1. A bird’s-eye-view projection (right) superimposes combined loadings of G-, C-, and A/U-ending codons. (**B**) A loadings plot of principal components 1 and 2 demonstrates discrimination of G- and C-ending codons (orange and pink, respectively) from A- and U-ending codons (blue and green, respectively) as features explaining the most variance. (**C**) Top 10 overrepresented KEGG pathways in each k-means cluster (*x*-axis; from Fig. S1) that have *P*_adj_ < 0.2. Numbers in parentheses represent the number of genes in each k-means cluster that mapped to a KEGG pathway.

It is known that pathogenicity islands, such as the LEE, possess different (and typically lower) GC content and codon usage than the rest of the genome (18). Indeed, we found that EHEC O157:H7 strain EDL933’s virulence-associated genes, and particularly LEE genes, have a striking bias for A/U-ending codons (Fig. 1). However, the functional consequences of this are unknown. Here, we induce virulence of EHEC O157:H7 strain EDL933 with the catecholamine norepinephrine (NE), the primary neurotransmitter of the sympathetic nervous system (3). Mesenteric organs, such as the small and large intestine, release more than half of all NE in the body, creating a gradient from the epithelial lining to the lumen (19). Gut pathogens, such as *Campylobacter*, *Salmonella*, and EHEC, respond to the NE gradient by upregulating chemotaxis and virulence pathways and increasing adherence to gut epithelial cells (19–22). In EHEC, NE acts through a complex signaling cascade involving two-component system histidine kinases QseC and QseE and response regulators QseB, QseF, and KdpE (3). However, some studies report that the actual signal being recognized is a metabolic byproduct of NE, 3,4-dihydroxymandelic acid (DHMA), which acts as a chemoattractant (20). Further, *Campylobacter* responds to NE despite lacking QseC, while in *Salmonella,* QseC and QseE are not required for NE-enhanced enteritis (22). These findings suggest that NE may stimulate virulence through multiple modalities. Using systems-level analyses combining tRNA modifications, tRNA isoacceptor abundance, and the proteome, we identified NE-induced changes in tRNA wobble modifications that correlate with selective translation of mRNAs enriched with A/U-ending codons. Together, these findings provide evidence that tRNA-mediated translational regulation can act as an additional layer of control over the LEE.

## RESULTS

### EHEC virulence-associated genes are biased for A/U-ending codons

To consider the possibility of codon-biased translation in the EHEC response to NE, we first assessed biased codon usage in the EHEC genome. We analyzed isoacceptor codon usage for all 5,356 genes to quantify over- or under-usage of codons relative to the genome average, with a *Z*-score calculated for each codon in each gene in the EDL933 genome, as previously described for other organisms (Data S1) (23–27). This analysis revealed that 3,224 genes significantly over- or underuse (*P* < 0.05) at least one codon. Genes and codons were then k-means clustered based on these codon *Z*-scores into five groups (Morpheus [28]) (Data S2). Indeed, clusters emerged comprising genes with similar codon usage patterns (Fig. S1). The *Z*-score data were then subjected to three-component principal component analysis (PCA) (Fig. 1A). Correlation loadings reveal that most variance is explained by the wobble base of the codon (Fig. 1B). Specifically, there is a divergence in genes that overuse G/C- versus A/U-ending codons (Fig. 1A top view). As demonstrated in Fig. 1A and Fig. S1, k-means Cluster 3 demonstrates a striking overuse of A/U-ending codons. By contrast, Clusters 1 and 5 overuse several C-ending codons, Cluster 2 overuses several G-ending codons, and Cluster 4 overuses specific G- and U-ending codons.

To gain understanding of what biological functions may be associated with the codon-biased gene clusters, we applied Bioconductor’s ClusterProfiler (29–32) KEGG pathway over-representation analysis to each k-means cluster (Fig. 1C). Several pathways showed statistically significant enrichment. In Cluster 1, many KEGG pathways related to active growth processes, including ribosome assembly, aerobic respiration/TCA cycle, glycolysis, and aminoacyl tRNA biosynthesis were enriched. In Cluster 2, virion-related genes were enriched, indicating that EHEC’s 18 prophage regions have a codon usage signature. Cluster 3, which is characterized by overuse of A/U- ending codons, is enriched in virulence-associated pathways, including T3SS genes. Notably, the entire LEE is in gene Cluster 3, in addition to both subunits of both Shiga toxins. While Clusters 4 and 5 both did not exhibit any statistically significant (*P*_adj_ <0.05) results, Cluster 4 showed slight enrichment of quorum sensing and flagellar motility-related genes.

PCA, clustering, and pathway analysis demonstrate clear evidence of biased codon usage among functionally related gene families. This highlights the potential for translational regulation through changes in tRNA wobble base modification status, creating a dynamic tradeoff between optimal translation of transcripts associated with different codon-biased biological functions. For instance, Cluster 1 contains genes encoding highly abundant housekeeping genes and ribosomal proteins that overuse primarily C-ending codons as well as other codons predicted to be decoded by highly abundant tRNAs under standard growth conditions (33). Under conditions in which the tRNA pool is instead optimized for efficient translation of A/U-ending virulence-associated genes, these housekeeping transcripts could be translated less favorably, enabling the cell to direct resources toward specific cellular programs.

It is worth noting that the observed codon usage bias in virulence genes is conserved among other pathogenic *E. coli* strains. Pathogenicity islands tend to exhibit lower GC content than the *E. coli* core genome and consequently have lower usage of G- and C-ending codons. As an example, the UPEC core genome GC content is roughly ~50%, whereas combined GC content of its pathogenicity islands I536, II536, III536, IV536, V536 is ~41% (9). Repeating our analysis with other *E. coli* strains, both pathogenic (ETEC H10407, UPEC UTI89) and commensal (MG1655), we consistently observed that highly abundant housekeeping genes tend to be biased for specific C-ending codons, whereas genes associated with adhesion, biofilm formation, and virulence tended to be biased for A/U-ending codons (Data S3).

### NE reprograms tRNA modifications but not the number of tRNA copies in EHEC

**Fig 2:**
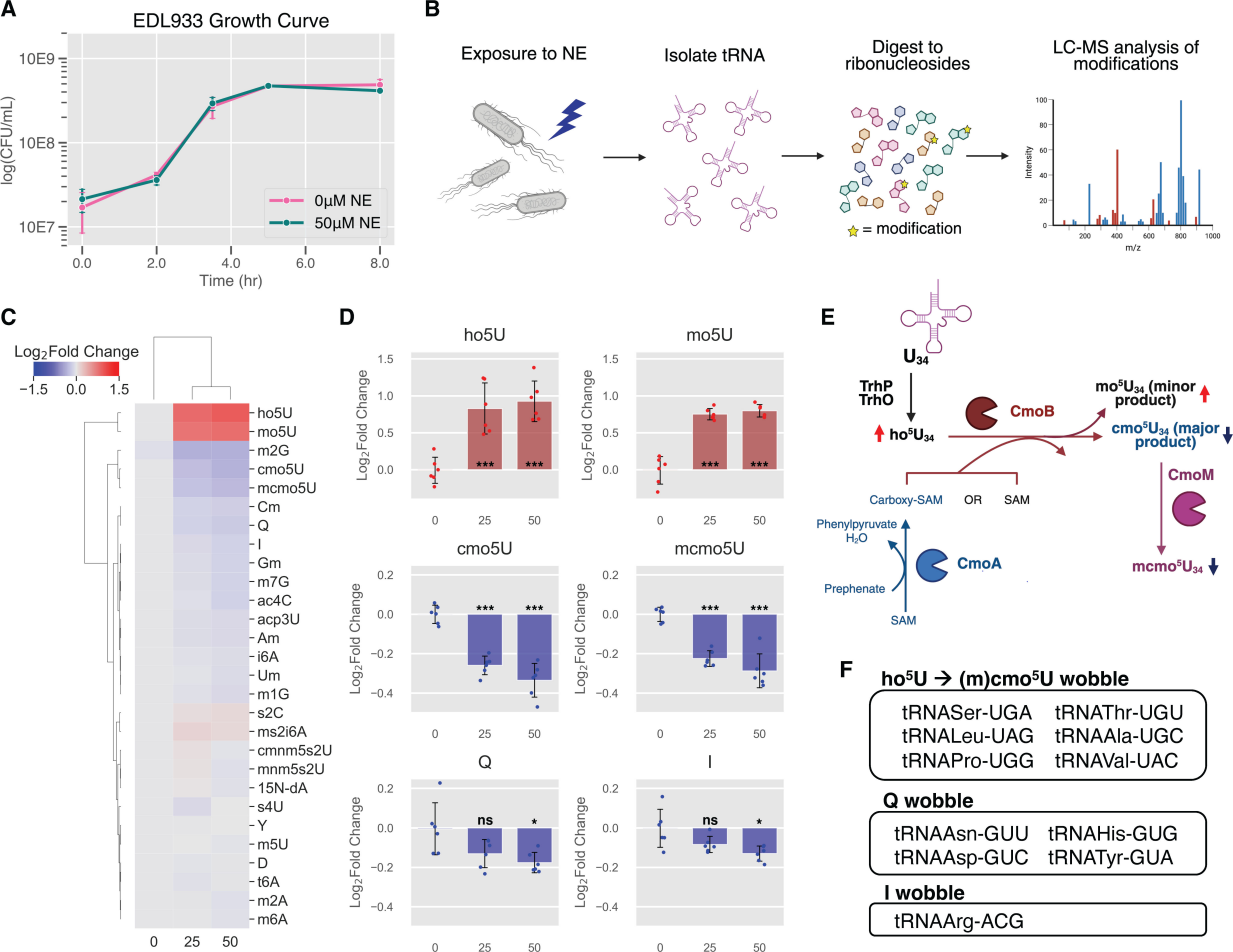
NE exposure mainly alters tRNA wobble modifications. (**A**) Growth curve of EHEC in serum-free low glucose DMEM ± 50 µM NE, reflecting three independent biological replicates. NE did not cause significant growth differences. (**B**) Experimental workflow: EHEC was grown ± NE and small RNA was extracted after 5 h. Small RNA was then digested to ribonucleosides that were analyzed by LC-MS. Image made with BioRender.com. (**C**) Heatmap showing log_2_(fold change) values of tRNA modifications in response to 25 and 50 µM NE. Values represent an average of six independent biological replicates across two different experiments. [^15^N_5_]-dA was included as an internal standard to control for day-to-day variance. (**D**) Several tRNA wobble modifications are changed significantly in response to a gradient of NE (*P* < 0.05, unpaired two-tailed Student’s *t*-test). Q, queuosine; I, inosine. (**E**) 5-Hydroxyuridine modification pathway. In response to NE, we see increases in intermediates/side products ho^5^U and mo^5^U and decreases in hypermodified forms of these modifications, cmo^5^U, and mcmo^5^U. Figure made with BioRender.com. (**F**) List of tRNA isoacceptors that contain wobble modifications altered by NE.

Given that genes associated with the infection process overuse A/U-ending codons, we next sought to test the links between NE exposure, reprogramming of the tRNA pool, and codon-biased translation of A/U-rich virulence genes. EHEC EDL933 was grown in low glucose DMEM with NE concentrations of 0, 25, and 50 µM, levels thought to be reached locally within the gut (34–36). Previous studies have shown that NE in this concentration range can stimulate bacterial growth in serum-based media, likely because NE promotes iron shuttling between transferrin molecules, thereby increasing iron availability for uptake by siderophores (37–39). Using DMEM with no serum supplementation, we do not observe significant growth differences, consistent with previous studies using serum-free media (Fig. 2A) (40). We thus performed NE stimulation experiments in the absence of serum to remove the confounding variable of NE-induced cell growth. After 5 h of growth (early stationary phase), small RNAs were extracted, digested to ribonucleosides, and subjected to LC-MS analysis to quantify changes in modification levels (Data S4; Fig. 2B) (41).

We observed several tRNA wobble modifications with statistically significant changes in response to a NE gradient, including several expected to contribute to A/U- versus G/C-ending codon biases in translation (Fig. 2C and D). 5-Hydroxyuridine modifications, which decorate the wobble-U of tRNAs that read from fourfold degenerate codon boxes (Fig. 2E and F), showed the largest changes. In *E. coli*, ho^5^U at position 34 is converted to mo^5^U or cmo^5^U by CmoB depending on the availability of co-factor carboxy-SAM, which is produced from SAM and prephenate by CmoA (Fig. 2E). In tRNAs with G at position 35, cmo^5^U34 is methylated to form mcmo^5^U34 by CmoM, which further stabilizes the wobble base (42). In response to NE, we observe increases in the precursors ho^5^U and mo^5^U and decreases in the hypermodified cmo^5^U and mcmo^5^U products, which could reflect a decrease in CmoA activity or a decrease in its substrate, prephenate. Both cmo^5^U and mcmo^5^U function to expand the decoding capacity of the tRNA and are critically important for reading G-ending codons (and C-ending codons, in the case of Ala, Pro, and Val) (42, 43). Without the (m)cmo^5^U modification, the tRNA is restricted to primarily the A-ending codon, with some affinity for the U-ending codon (43).

Wobble modifications queuosine (Q) and inosine (I) also significantly decreased at 50 µM NE. Q is a non-essential modification present on tRNAs with GUN anticodons (Fig. 2F) and has been shown to affect translational fidelity and bias for C- versus U-ending codons. However, the direction of this bias is ambiguous and seems to depend on sequence context (44–46). I is a wobble modification exclusively present on tRNA-ArgACG in *E. coli* (47). It serves to expand the decoding capacity to include C- and A-ending codons, in addition to the expected U-ending codon based on A34 of the tRNA (47). Aside from strictly wobble modifications, m^2^G, which is found on *E. coli* rRNA as well as position 26 of tRNAs, was decreased in response to NE. m^2^G reduction has been observed in the past in response to oxidative stress (48, 49).

**Fig 3:**
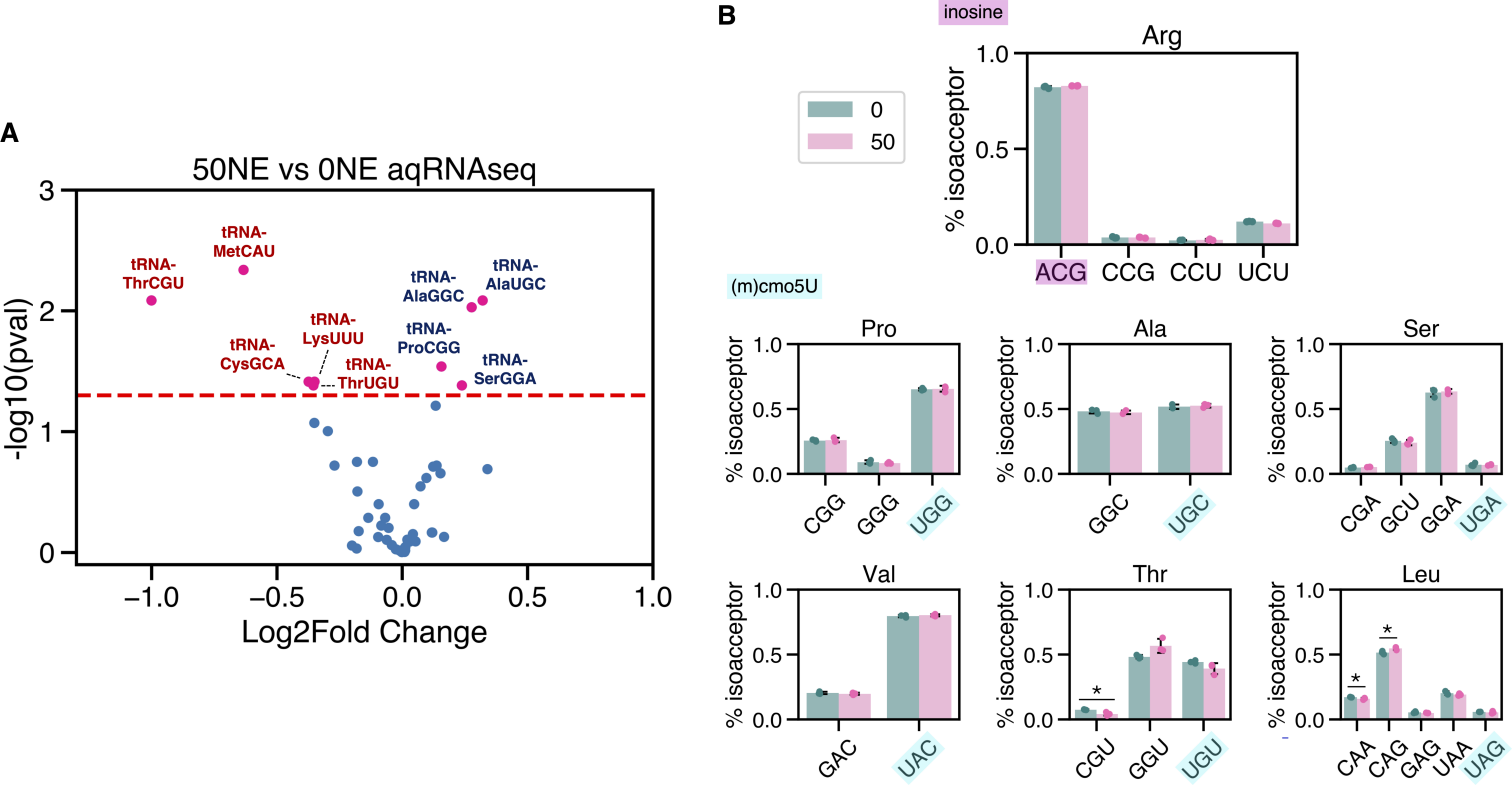
Absolute quantitative RNA-sequencing (AQRNA-seq) reveals minimal changes in tRNA levels and tRNA isoacceptor ratios. (**A**) Volcano plot of tRNA abundance changes relative to the whole tRNA pool. Fold changes and adjusted *P* values were calculated with DESeq2 (52). (**B**) tRNA isoacceptor ratios for amino acids read by inosine and (M)cmo^5^U modified tRNAs, showing minimal changes between control and 50 µM NE-exposed samples. *P* values were calculated with an unpaired two-tailed Student’s *t*-test, with * designating *P* < 0.05. Anticodons that are highlighted are expected to have the specified wobble modification.

The large changes in tRNA wobble modifications raised the question of whether there were changes in levels of specific tRNAs carrying those modifications. We performed absolute quantitative RNA sequencing (AQRNA-seq), a technique for quantitative comparison of tRNA levels within a population (Data S5) (50, 51). Here, we asked if NE stimulation caused changes in tRNA levels that correlated with changes in modification levels. As a whole, a few tRNAs showed small but statistically significant changes—notably, Thr tRNAs were slightly decreased relative to the whole tRNA pool, whereas Ala tRNAs were slightly increased relative to the whole tRNA pool (Fig. 3A). However, the ratios of tRNA isoacceptors for Thr, Ala, Ser, Val, Pro, and Leu (containing cmo^5^U, mcmo^5^U) as well as Arg (containing I) were unchanged by 50 µM NE exposure (Fig. 3B). We define the ratio of tRNA isoacceptors (% isoacceptors in Fig. 3B) to be the percentage of reads mapped to tRNAs with a specified anticodon, relative to all tRNAs for that amino acid. For example, tRNA-Pro-UGG (modified with cmo^5^U) represents ~70% of all proline tRNAs (Fig. 3B). In addition, Asn, Asp, His, and Tyr codons are each read by a single queuosine-modified tRNA isoacceptor, and none of these four tRNAs exhibited statistically significant changes. Together, these observations suggest that the measured changes in (m)cmo^5^U, Q, and I are primarily driven by changes in the modification status of a fixed population of tRNAs and not by changes in the levels of the modified tRNAs.

While we do not observe large changes in tRNA abundance, it is possible that NE exposure alters tRNA aminoacylation status. Here, we note that aminoacyl-tRNA biosynthesis is among the most downregulated pathways following NE exposure (Fig. 5D). While the modifications highlighted above have not been explicitly linked to altered aminoacylation kinetics, this is the case for certain U34 modifications, such as those containing the s^2^U moiety (53). There are also not obvious links between these particular modifications and tRNA structural stability, although wobble modifications can influence tRNA function through changes in anticodon loop folding as well as protecting tRNAs from ribonuclease cleavage, as is the case for Q modification (54).

**Fig 4:**
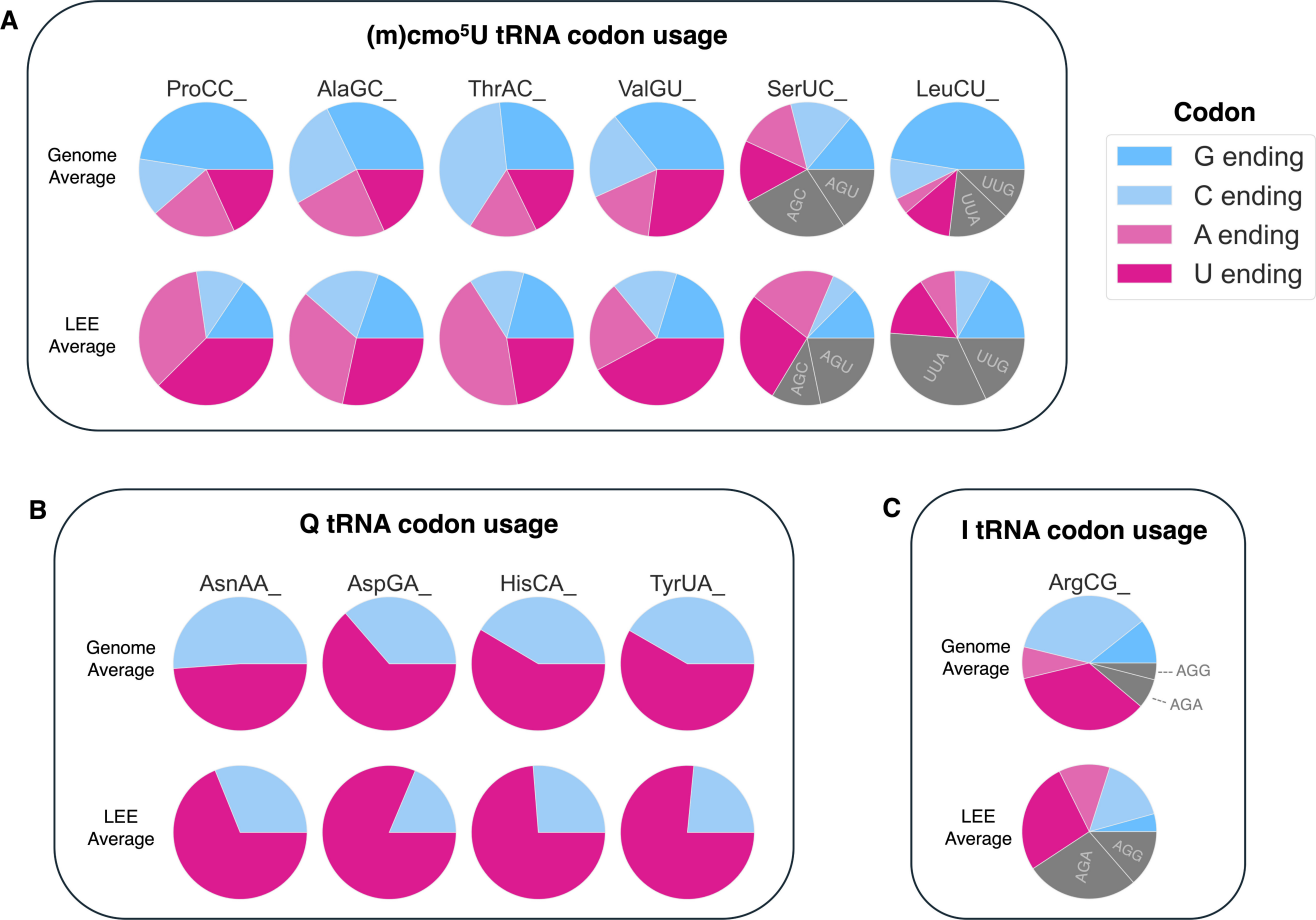
Codon usage comparison between LEE genes and genome averages. (**A–C**) Light and dark pink represent A- and U-ending codons, respectively, while dark blue and light blue represent G- and C-ending codons, respectively. Gray codons are alternative codons that are not read by the tRNA with the specified wobble modification. Results are shown for codons read by (**A**) (m)cmo^5^U-modified tRNAs, (**B**) Q-modified tRNAs, and (**C**) I-modified tRNAs. Codon frequency data comparisons between LEE and non-LEE genes, including statistical analyses, are in Table S1.

Figure 4 pulls together the underuse of G/C-ending codons in LEE genes (Fig. 1; Table S1) and the tRNA isoacceptors possessing wobble modifications that are significantly reduced by exposure to NE (Fig. 2). The correlation that emerges is that NE causes a decrease in the proportion of tRNAs bearing wobble modifications that are critical for reading of G/C-ending codons, which are underused in NE-induced LEE virulence genes. The loss of wobble (m)cmo^5^U, Q, and I modifications may bias these tRNAs to read A/U-ending codons enriched in LEE genes (Table S1). We thus hypothesized that these tRNA modification changes would pair with codon biases apparent in NE-induced changes in the steady-state levels of proteins.

### Changes in tRNA modifications correlate with codon-biased translation in NE-exposed EHEC

**Fig 5:**
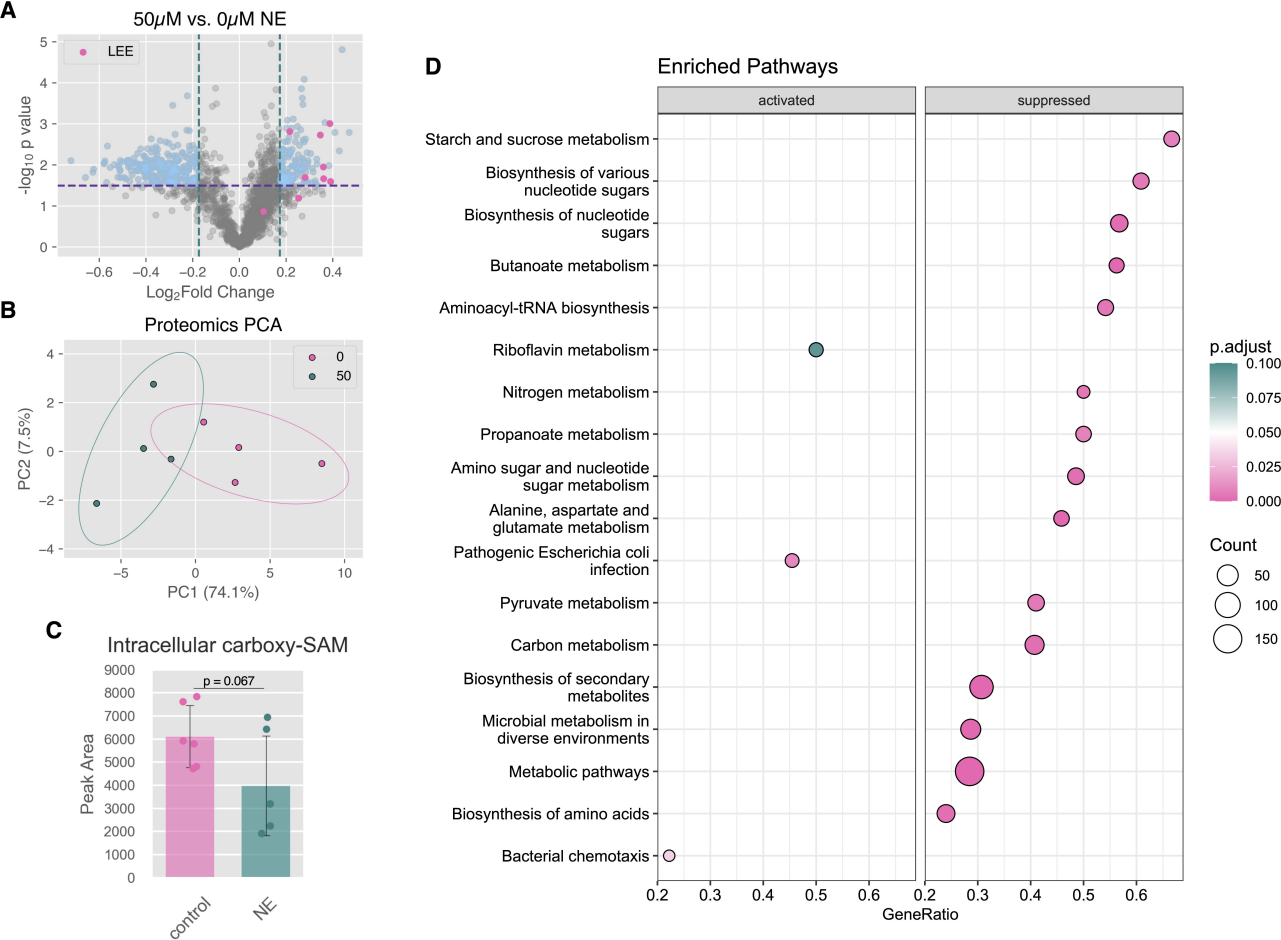
NE exposure reprograms the EHEC proteome. (**A**) Volcano plot demonstrating differential protein expression in response to NE. Threshold shown in purple represents a Benjamini-Hochberg adjusted *P*-value of 0.1. Vertical lines represent ±1 standard deviation from the mean. Detected LEE proteins are shown in pink. (**B**) Principal component analysis of proteomics data, showing separation of NE exposed samples along PC1. Ellipses represent 95% confidence intervals. (**C**) NE exposure reduces intracellular levels of carboxy-SAM, as measured by log_2_(fold change) in mass spectrometry peak areas normalized by cell number (*P* = 0.067, unpaired two-tailed Student’s *t*-test). (**D**) KEGG pathway enrichment results of proteomics data obtained with clusterProfiler package, showing activation of virulence and chemotaxis-associated pathways and suppression of diverse metabolic pathways in response to NE. The top 15 differentially regulated pathways are shown.

To test the hypothesis that NE-induced changes in tRNA wobble modifications are correlated with codon-biased shifts in the proteome, we used tandem mass tag (TMT) proteomics to quantify 1,810 proteins with at least 2 unique peptides across all samples (Data S6). Samples exposed to 50 µM NE showed a distinct proteome, with changes in 366 proteins exhibiting a Benjamini-Hochberg adjusted *P*-value < 0.1 and a magnitude of log_2_(fold change) greater than 1 standard deviation (Fig. 5A and B). Consistent with expectations set by prior literature, LEE proteins were among the most upregulated proteins in response to NE (3). Further, KEGG pathways “Pathogenic *Escherichia coli* infection” and “Bacterial chemotaxis” were activated, while a multitude of metabolic pathways were suppressed (Fig. 5D).

**Fig 6:**
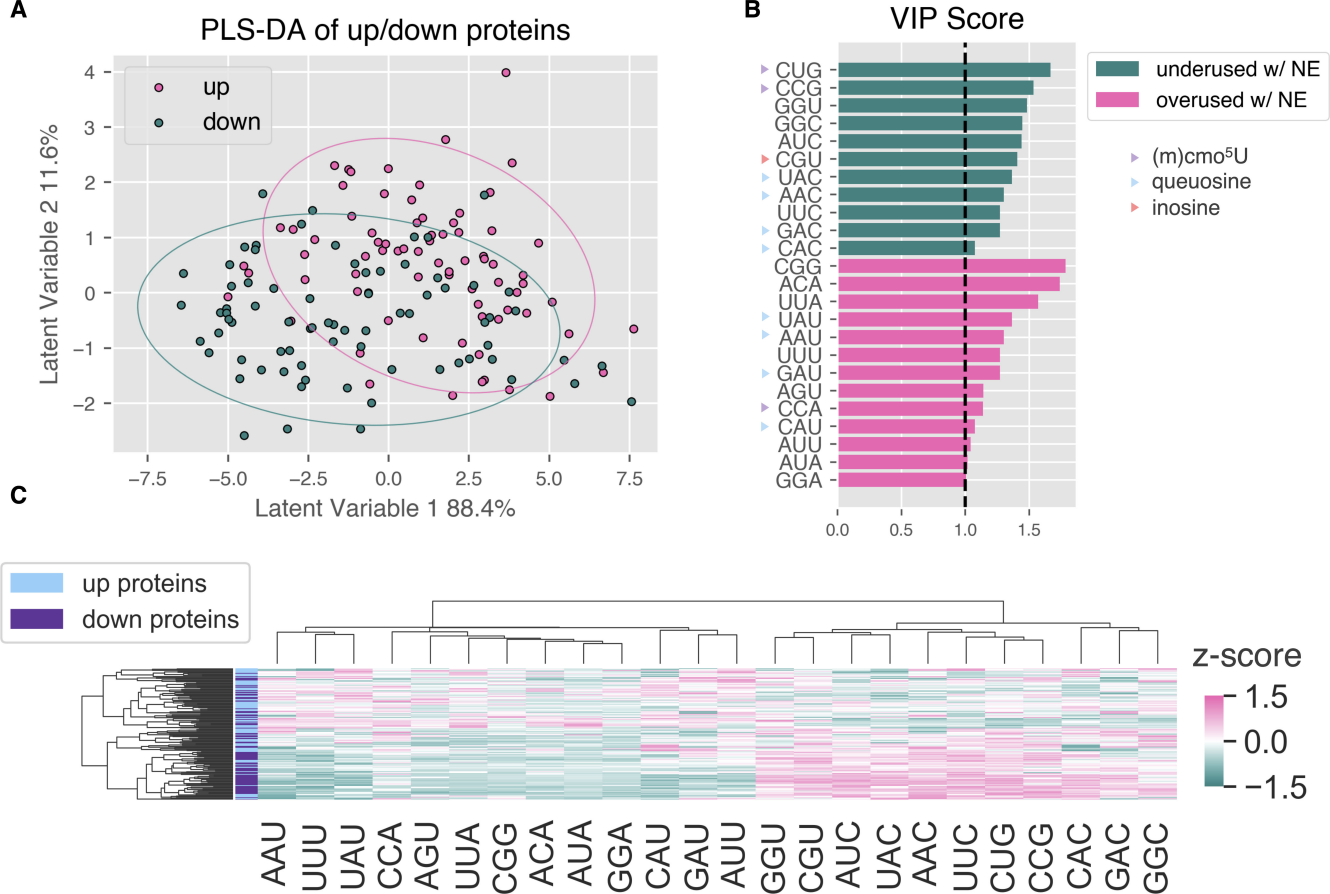
Partial least squares classification of protein up- or downregulation based on codon usage patterns. (**A**) Scores plot of upregulated (pink) and downregulated (teal) proteins. Ellipses show 95% confidence intervals. (**B**) Codons with VIP scores >1, with indications of whether they are overused following NE exposure (pink) or underused (teal). Codons marked with triangles are read by tRNAs with the specified wobble base modifications. (**C**) Heatmap of top 75 up- and downregulated proteins, hierarchically clustered by codon usage *Z*-scores (Euclidean distance metric). Included codons have VIP scores >1.

Proteins were ranked by the product of signed log_2_(fold change) and −log_10_(*P*-value). Unsupervised clustering based on codon *Z*-scores of the top and bottom 75 proteins revealed two main clusters—one with primarily “up” proteins, and one with primarily “down” proteins (Fig. S2). To more rigorously assess which codons were predictive of up- and downregulation at the protein level, we performed partial least squares discriminant analysis (PLS-DA) using the proteomics and codon usage data sets (Data S1 and S6). From this model, we identified codons with a variable importance in projection (VIP) score greater than 1 (Fig. 6A and B).

Underused codons with VIP scores >1.5 include LeuCUG and ProCCG, two G-ending codons read through wobbling by tRNAs modified with cmo^5^U (Fig. 6B and C). This is consistent with the observation that cmo^5^U decreases upon NE exposure. In EDL933, CCG and CUG are significantly underused in LEE genes (*P*_CCG_ = 5.8E−12, *P*_CUG_ = 4.3E−17, unpaired Student’s two-tailed *t*-tests), comprising nearly half of all proline and leucine codons, respectively, while representing less than a quarter of proline/leucine codons in LEE genes (Fig. 4A; Table S1). Proteins with mRNAs heavily overusing both LeuCUG and ProCCG are particularly impacted by a reduction in the cmo^5^U modification, since under-modified tRNAs are primarily restricted to the A-ending codon. Having a fully modified tRNA-ProUGG is expected to be particularly important for efficient decoding of G/C-ending proline codons, since it is the most abundant proline isoacceptor in EHEC EDL933 which can read all four proline codons only when modified with cmo^5^U (Fig. 3B).

Additionally, all eight codons read by the four Q-modified tRNAs had a VIP score >1, and all the U-ending codons were overused, while all the C-ending codons were underused upon NE exposure (Fig. 5B). Our observation of a decrease in Q is at odds with a previous study showing that Q deficiency in *E. coli* DH10B Δ*queF* causes slight translational bias for C-ending codons (14). However, Q-deficient *E. coli* MG1655 Δ*tgt* versus wild-type showed mild bias for U-ending codons AsnAAU, AspGAU, and TyrUAU (HisCAU/C bias was not easily resolved) (44). These contradictions reflect the complexity of the role of Q in C/U-ending codon bias, with some studies reporting that Q has no contribution to codon bias, whereas others report the direction of the bias being different in different organisms, in different contexts, and for different codons (46, 55–57).

We next examined the proteomics data to determine whether changes in writer enzyme abundance could account for the observed differences in wobble base modifications (Fig. S3). At the 5-h time point, TadA levels showed no statistically significant change, consistent with the modest decrease in I. In contrast, the writer enzyme Tgt exhibited a small but significant decrease, which may explain the small reduction in Q. For the 5-hydroxyuridine-based modifications, the coordinated increase in ho⁵U and mo⁵U and decrease in cmo^5^U and mcmo^5^U suggest altered CmoA activity (Fig. 2C and E). Although proteomic data indicated reduced CmoA abundance, this trend did not reach statistical significance. We therefore considered whether limited availability of prephenate—the substrate for CmoA and a precursor for aromatic amino acid biosynthesis—might also contribute.

Previous studies have shown that (m)cmo^5^U modification levels are intimately linked to changes in metabolic fluxes, including aromatic amino acid biosynthesis (the shikimate pathway) and, in *Pseudomonas aeruginosa*, phenazine biosynthesis (58, 59). Supporting this idea, we observed decreased levels of bifunctional chorismate mutase/prephenate dehydrogenase PheA (*P*_adj_ = 0.08), which generates prephenate as part of the shikimate pathway, as well as smaller decreases in other shikimate pathway-associated proteins (Fig. S3). These findings were corroborated by reverse transcription quantitative PCR (RT-qPCR), where we observed that *cmoA* transcript abundance was unchanged, while *pheA* showed a nearly twofold decrease in transcript abundance (Fig. S4). We further performed LC-MS/MS analysis of intracellular carboxy-SAM in the presence of NE during log-phase growth (3.5 h), which showed a decrease upon NE exposure, although this was not statistically significant (*P* = 0.067) (Fig. 5C). Together, these results suggest that changes in 5-hydroxyuridine modification levels are not due to changes in CmoA abundance but instead may reflect NE-induced metabolic changes that limit prephenate availability.

## DISCUSSION

While it is known that the EHEC LEE pathogenicity island exhibits distinct codon usage compared to the rest of the genome, the functional consequences of this bias remain poorly understood, particularly under conditions that induce T3SS expression. We therefore tested whether EHEC modifies its translation machinery to accommodate the increased demand for decoding A/U-ending codons enriched in LEE genes. Upon T3SS induction by NE exposure, we observed remodeling of the tRNA pool, characterized by reduced levels of several wobble modifications required for efficient decoding of G/C-ending codons. At the proteome level, this corresponded with a global shift: downregulated proteins were disproportionately enriched in G/C-ending codons reliant on these modifications, whereas upregulated proteins tended to underuse them.

Our findings build upon a growing body of evidence that dynamic changes in the tRNA epitranscriptome play an important role in microbial virulence (13–15). Notably, the contributions of certain tRNA modification enzymes to virulence may be overlooked by traditional transposon-based genetic screens, as certain tRNA modifications are essential (for example, I), whereas the absence of others can impair growth (for example, 5-hydroxyuridine modifications) (60). In *E. coli*, Q is nonessential, its absence does not affect growth, and its presence has been linked to biofilm formation and resistance to oxidative stress, heat shock, UV irradiation, and heavy metal stress in *E. coli* and closely related species (14, 44). The Tgt enzyme has also been identified as a hit in a rabbit colon colonization transposon screen (61).

It remains to be answered how NE exposure induces these changes in the tRNA pool. Carboxy-SAM-dependent 5-hydroxyuridine modifications changed the most. Given that prephenate, a precursor to aromatic amino acid biosynthesis, is a critical CmoA substrate for carboxy-SAM generation, and that NE itself is derived from the aromatic amino acid tyrosine, we speculate that there could be interesting links between NE exposure and feedback inhibition of the shikimate pathway. Additionally, other studies have shown that in both EHEC and non-pathogenic *E. coli*, NE exposure reduces metabolites of the aromatic amino acid tryptophan and also causes downregulation of the tryptophanase TnaA (62). One such tryptophan metabolite is indole, which serves as a microbiota-derived signal to suppress LEE gene expression in the intestinal lumen as opposed to the gut epithelial surface (6). Our own data suggest that 5 h after NE exposure, TnaA levels are decreased [log_2_(fold change) = −0.28, *P*_adj_ = 0.13], albeit not significantly (Data S6). These observations suggest a broader link between NE exposure, aromatic amino acid flux, and the availability of precursors required for 5-hydroxyuridine modification of tRNA.

Our findings support a model in which EHEC employs translational regulation through remodeling of the tRNA epitranscriptome to bias protein synthesis toward codon patterns enriched in virulence genes. Importantly, NE also triggers a well-documented transcriptional response, and prior studies have shown that LEE mRNA levels increase under these conditions (3, 34, 40), which is consistent with a coordinated regulation of gene expression at the levels of both transcription and translation. In response to NE, EHEC both elevates LEE transcript abundance and reprograms its tRNA pool, thereby promoting efficient translation of these virulence mRNAs and others with similar codon usage. This work highlights translational regulation via the tRNA epitranscriptome as a previously underappreciated layer of virulence control, expanding the paradigm of how bacterial pathogens coordinate gene expression during infection.

## MATERIALS AND METHODS

### Strains and culture conditions

Each biological replicate was derived from a single colony of enterohemorrhagic *E. coli* strain EDL933 (ATCC 43895), grown aerobically overnight in LB media. For NE exposure experiments, low glucose DMEMsupplemented with HEPES and pyruvate was used (ThermoFisher catalog no. 12320032). LB overnight cultures were spun down (3,700 × *g*, room temperature), resuspended in low glucose DMEM, and diluted 1:100 in low glucose DMEM, with or without 50 µM norepinephrine bitartrate salt (NE;, Sigma A0937). Cultures were grown aerobically for 5 h, shaking 180 rpm at 37°C.

### Small RNA extraction

Unless stated otherwise, the following steps were performed at ambient temperature. After growth, cells were harvested by centrifugation for 4 min at 3,700 × *g*. After removal of the supernatant, cell pellets were resuspended in Trizol (1:1 ratio with culture volume) and mixed well by vortexing for 15 s. After 5 min of incubation, 0.2 mL of chloroform per 1 mL of Trizol reagent was added to each sample, followed by shaking vigorously by hand for 15 s. After 3-min incubation, samples were centrifuged at 12,000 × *g* for 15 min at 4°C. The aqueous phase was isolated and brought to a concentration of 30% ethanol to precipitate large RNAs, then applied to a spin column from the Purelink miRNA isolation kit (ThermoFisher catalog no. K157001). After centrifugation at 13,000 × *g* for 1 min, the flowthrough containing small RNAs was collected, brought to an ethanol concentration of 70%, and applied to a second spin column. Small RNAs were washed twice with wash buffer from the Purelink kit, then spun dry for 2 min at 13,000 × *g* to remove excess wash buffer, followed by elution in sterile, non-DEPC treated, nuclease-free water. Small RNA concentration was quantified by Nanodrop and small RNA purity and quality was assessed by Agilent 2100 Bioanalyzer Nanochip.

### RNA hydrolysis

One microgram of RNA was digested in a 50-µL reaction containing 5 mM Tris-HCl (pH 8.0), 2.5 mM MgCl_2_, 0.1 mg/mL coformycin (adenosine deaminase inhibitor), 0.1 mM deferoxamine (antioxidant), 0.1 mM BHT (antioxidant), 0.083U/µL benzonase, 0.1 U/µL calf intestinal alkaline phosphatase, 0.003 U/µL phosphodiesterase I, and 50 nM of [^15^N]dA as an internal standard to adjust for day-to-day variance. The digestion mixture was incubated at 37°C for 6 h. Then, the mixture was spun down at 3,000 × *g* for 10 min at 4 °C, and the supernatant was collected and transferred to HPLC vials.

### LC-MS/MS analysis of ribonucleoside modifications

Digested ribonucleosides were analyzed by chromatography-coupled triple-quadrupole mass-spectrometry (LC-MS/MS). Approximately 600 ng of hydrolysate for each sample was analyzed by three technical replicate injections of 200 ng. Briefly, a Waters ACQUITY BEH C18 column (50 × 2.1 mm inner diameter, 1.7-μm particle size) was used with an Agilent 1290 HPLC system at 25°C, 0.3 mL/min flow rate, in a gradient of buffer A (0.02% formic acid in water) and buffer B (0.02% formic acid in 70% acetonitrile) (Table S2). Retention times of modified ribonucleosides were determined by synthetic standards. The HPLC was coupled to an Agilent 6495c triple quadrupole mass spectrometer with an electrospray ionization source in positive mode with 200°C dry gas temperature, 11 L/min gas flow, 20 psi nebulizer pressure, 300°C sheath gas temperature, 12 L/min sheath gas temperature, 3,000 V capillary voltage, and 0 V nozzle voltage. Product ions were detected using dynamic multiple reaction monitoring (MRM) mode, with collision energies optimized for maximum sensitivity detection of each target ion (Table S3). Peak areas were normalized to the sum of 260 nm UV signals of the four canonical nucleotides, as measured by an in-line UV detector. LC-MS/MS data for the ribonucleoside analyses are presented in Data S4.

### Absolute quantitative RNA sequencing (AQRNA-seq)

AQRNA-seq was conducted to measure changes in tRNA isoacceptor levels upon NE exposure. Briefly, 75 ng of purified small RNA per sample was used to construct cDNA libraries using the protocol described in previous work, with some revisions (50, 51). These included (i) the use of a high-processivity reverse transcriptase that resists fall-off in the presence of diverse tRNA modifications and (ii) an exonuclease I digestion step after reverse transcription that removes excess reverse transcription primers, thereby mitigating primer-dimer formation. These protocol changes remove the need for AlkB demethylation and gel purification. Constructed libraries were submitted to the MIT BioMicro Center, where they underwent quality assessment using the AATI Fragment Analyzer (Agilent) and were quantified by the LightCycler 480 Real-Time PCR System (Roche). Libraries were sequenced on the Illumina MiSeq platform using the v3 reagent kit (Illumina) to obtain 75-bp paired-end reads. Raw sequences were processed with an in-house pipeline to estimate tRNA abundance. Processed AQRNA-seq data is available as Data S5.

### Proteomics sample preparation

Cell pellets were resuspended in a lysis buffer containing 50 mM TEAB, 1% sodium deoxycholate, and Roche cOmplete EDTA-free protease inhibitors (no. 11873580001). After resuspension, samples were heated at 80°C for 5 min on a heat block, then immediately cooled on ice. Samples were then vortexed for 3 × 30 s, resting on ice between vortexes. Next, samples were subjected to tip sonication on an ice bath (eight pulses at a 50× duty cycle, rested on ice, followed by a second eight pulses). Debris was spun down at 4,000 × *g* for 10 min 4°C, and the supernatant was transferred to a protein LoBind tube. Protein was quantified by BCA assay using BSA as a standard. Protein for each sample (120 µg) was reduced, alkylated, trypsinized, and washed free from impurities and detergents using an S-trap mini kit (Protifi K02-mini-10), following kit instructions. Eluted peptides were dried down and given to the MIT Koch Proteomics core for desalting and 11-plex TMT labeling (ThermoFisher). Dried, labeled peptides were resuspended in 10 mM TEAB and fractionated on an Agilent 1100 HPLC with 10 mM TEAB (Buffer A) and 10 mM TEAB in 99% Acetonitrile (Buffer B) (Table S4). A total of 80 fractions were collected every minute starting at minute 10. Fractions distributed equally across time points were combined to make eight fractions for analysis (Table S5).

### Nano-LC-Orbitrap analysis of the EHEC proteome

TMT-labeled, pooled, and fractionated peptides were analyzed on a ThermoFisher EASY-nLC 1000 using a precolumn and a self-packed 5-mm tip analytical column (15 cm of 5 mm C18, New Objective) over a 146-min gradient (Table S6). The pump was interfaced to a QExactive Orbitrap mass spectrometer (ThermoFisher). Mass spectrometric scan parameters were 70,000 resolution across 350–2,000 *m/z*, AGC target of 3e6, and maximum IT of 200 ms. The top 10 precursor ions of the full scan MS in each cycle were analyzed by MS/MS using a normalized collision energy of 34% and a dynamic exclusion of 30 s. Raw mass spectral files were searched in Proteome Discoverer (ThermoFisher) using the Sequest database. Parameters for the search included a precursor mass tolerance of 10 ppm, a fragment ion mass tolerance of 0.6 Da, a maximum of two missed cleavages of trypsin, fixed modifications of cysteine carbamidomethylation and lysine TMT label, and dynamic modification of methionine oxidation. Our analysis included 1,810 high-confidence proteins that were found in all samples and identified with at least two unique peptides >6 amino acids in length. Reporter ion intensities were log-transformed and quantile normalized prior to fold change calculation. Full proteomics data are available as Data S6.

### Intracellular carboxy-SAM measurement

Intracellular carboxy-SAM was measured using a method adapted from Kim et al. (63). Overnight cultures were inoculated 1:500 into fresh serum-free low glucose DMEM media ± 50 µM NE and grown at 37°C, shaking at 180 rpm for 3.5 h. A 10-µL aliquot of each culture was serially diluted and plated for CFU counting. Cultures were then pelleted at 3,700 × *g* for 5 min. The supernatant was removed, and the cell pellet was flash frozen and stored at −80°C. Frozen cell pellets were resuspended in ice-cold extraction buffer composed of 2:2:1 methanol:acetonitrile:water with 0.1% formic acid at a ratio of 4 × 10^8^ CFU per 1 mL. Samples were vortexed well to mix, incubated on ice for 30 min, and thoroughly vortexed again. Samples were centrifuged at 13,000 × *g* and 4°C for 20 min to precipitate protein. Supernatant from each sample (200 µL) was transferred to a clean 500-µL tube, dried via SpeedVac, and stored at −80°C until ready for mass spectrometry analysis.

A standard of carboxy-SAM was chemically synthesized as described in Method S1. LC-MS/MS analysis of carboxy-SAM in EHEC lysates was carried out as described in Method S2.

### Codon analytics

Gene-specific and genome-based codon analytic measures were made using the *Escherichia coli* O157:H7 str. EDL933 genome (GenBank: AE005174.2) using methods previously described (24–27). Briefly, 5,356 EHEC genes were analyzed from start to stop codon to count the number of each of 64 codons, with corresponding values used to generate gene-specific isoacceptor codon frequencies and *Z*-scores. Genome-based averages were also generated using the values for the 5,356 genes. *Z*-score data are available as Data S1.

### Data mining, informatics, and multivariate statistics

PCA and PLS-DA analyses were performed in Python using the sklearn package (v0.0) (64). K-means clustering based on codon usage data were performed using the Broad Institute’s Morpheus software (28). KEGG pathway overrepresentation tests and enrichment analyses were performed in R using Bioconductor’s clusterProfiler package (v4.14.6) (30–32). AQRNA-seq data were analyzed with R’s DEseq2 package (v1.46.0) (52). Proteomics data were log transformed, quantile normalized, analyzed for differential expression, and subjected to Students’ two-tailed *t*-tests in Python using numpy (v1.21.6) (65) and scipy.stats (v1.7.3) (66) packages. *P*-values from AQRNA-seq and proteomics were multiple hypothesis corrected by the Benjamini-Hochberg method.

## Data Availability

Raw data have been deposited in public repositories: AQRNA-seq data, GEO accession GSE309283; proteomics data, PRIDE accession PXD068911. All other processed data are included in supplemental files.

## SUPPLEMENTAL MATERIAL

Data S1A (1-2692) msystems.01418-25-s0001.xlsx Calculation of gene-specific codon usage *Z*-scores for genes 1–2,692.

Data S1A (2693-5356) msystems.01418-25-s0002.xlsx Calculation of gene-specific codon usage *Z*-scores for genes 2,693–5,356.

Data S1B msystems.01418-25-s0003.xls Gene-specific codon usage *Z*-scores.

Data S2 msystems.01418-25-s0004.xls K-means gene clustering based on codon usage and overrepresented KEGG pathways.

Data S3 msystems.01418-25-s0005.xlsb Codon usage *Z*-scores for three *E. coli* strains.

Data S4 msystems.01418-25-s0006.xls LC-MS/MS analysis of ribonucleosides.

Data S5 msystems.01418-25-s0007.xls AQRNA-seq analysis of tRNA levels.

Data S6 msystems.01418-25-s0008.xls Proteomics fold change data.

Supplemental Information msystems.01418-25-s0009.docx Figures S1 to S6, Tables S1 to S5, Methods S1 to S3, and captions for Data S1 to S6.

ASM does not own the copyrights to Supplemental Material that may be linked to, or accessed through, an article. The authors have granted ASM a non-exclusive, world-wide license to publish the Supplemental Material files. Please contact the corresponding author directly for reuse.
