# Supplementary material for: Enterohemorrhagic Escherichia coli O157:H7 responds to norepinephrine gradients by tRNA reprogramming and codon-biased translation of virulence genes
Source: mSystems. 2026 Apr 22;11(5):e01418-25. doi: 10.1128/msystems.01418-25 (PMC13185646; doi:10.1128/msystems.01418-25)
Supplement: Supplemental Information — Figures S1 to S6, Tables S1 to S5, Methods S1 to S3, and captions for Data S1 to S6. [file msystems.01418-25-s0009.docx]

**Supplementary Information**

**“Enterohemorrhagic *Escherichia coli* O157:H7 responds to norepinephrine gradients by tRNA reprogramming and codon-biased translation of virulence genes”**

Abigail E. McShane, Chi-Kong Chan, Ruixi Chen, Michael S. DeMott,

Thomas J. Begley, Peter C. Dedon

**Supplementary Figures**

Figure S1: K-means clustering the EDL933 genome based on codon usage

Figure S2: Top 75 up- and down-regulated proteins cluster by codon usage

Figure S3: Proteomics fold-change data of proteins predicted to affect levels of queuosine, hydroxyuridine, and inosine

Figure S4: RT-qPCR of shikimate-pathway associated genes

Figure S5: Extracted ion chromatograms obtained from LC-MS/MS analysis of carboxy-SAM

Figure S6: MS/MS spectrum obtained from collision-induced dissociation of the carboxy-SAM ion at *m/z* 443.1340 and predicted fragmentation of carboxy-SAM

**Supplementary Tables**

Table S1: Buffer gradient used in the LC-MS/MS of ribonucleosides

Table S2: Parameters for LC-MS/MS analysis of nucleosides

Table S3: Peptide fractionation gradient for proteomics

Table S4: Fractionated peptide pooling scheme

Table S5: Proteomics nLC gradient

**Supplementary Methods**

Method S1: Chemical synthesis and characterization of carboxy-SAM

Method S2: LC/MS-MS analysis of carboxy-SAM

Method S3: RT-qPCR of shikimate pathway associated genes

**Supplementary Data** (separate Excel spreadsheets)

***Supplementary Data 1A,B:*** Gene-specific codon usage Z-score data (three files).

***Supplementary Data 2***: K-means gene clustering based on codon usage and overrepresented KEGG pathways.

***Supplementary Data 3***: Codon usage in three *E. coli* strains

***Supplementary Data 4***: LC-MS/MS analysis of ribonucleosides.

***Supplementary Data 5***: AQRNA-seq analysis of tRNA levels.

***Supplementary Data 6***: Proteomics fold-change data.

**Figure S1. K-means clustering the EDL933 genome based on codon usage.** Every gene in the EDL933 genome (rows) was assigned a set of z-scores indicating whether a codon was over- or under- used relative to the genome mean (columns). Note that stop codons, as well as methionine ATG and tryptophan UGG, were excluded from this analysis. Genes and codons were both k-means clustered into 5 groups using the Jaccard distance metric. Assigned clusters are indicated with blue (genes) and purple (codons) along the heatmap axes. Codon columns are also color-coded according to the third codon base, demonstrating that codons cluster by this base.

**Figure S2. Top 75 up- and down-regulated proteins cluster by codon usage.** Proteins were ranked by the product of signed log_2_(fold-change) and -log10(p-value). The top and bottom 75 proteins from this list were hierarchical clustered based on codon usage patterns (using average Euclidean distance as a clustering metric).

**Figure S3. Proteomics fold-change data of proteins predicted to affect levels of queuosine (pink), hydroxyuridine (teal), and inosine (purple).** For queuosine, writer enzyme Tgt is slightly reduced but with high statistical significance. CmoA, the enzyme that generates carboxy-SAM for (m)cmo^5^U modification, is down but not significantly. PheA, a bifunctional chorismate mutase, is down, suggesting that the pool of prephenate, CmoA’s substrate, may be depleted in response to NE. Levels of the writer enzyme for inosine, tadA, are not significantly changed at this 5 h timepoint.

**Figure S4.** Transcript levels were normalized to the housekeeping gene gyrA, with gapA included as a secondary housekeeping control. Shown are fold-changes in transcript abundance for EHEC exposed to 50 µM versus 0 µM norepinephrine (NE) following 5 h of growth at 37 °C with shaking at 180 rpm in low-glucose DMEM. No significant changes in transcript abundance were observed for the writer enzyme cmoA or for the tyrosine-sensitive DAHP synthase aroF. In contrast, the tryptophan-sensitive DAHP synthase aroH exhibited a small but statistically significant decrease in transcript abundance. The bifunctional chorismate mutase/prephenate dehydrogenase pheA, which generates prephenate, showed a nearly two-fold, statistically significant decrease in expression. These data support the hypothesis that the observed changes in 5-hydroxyuridine modifications could result from reduced prephenate availability.


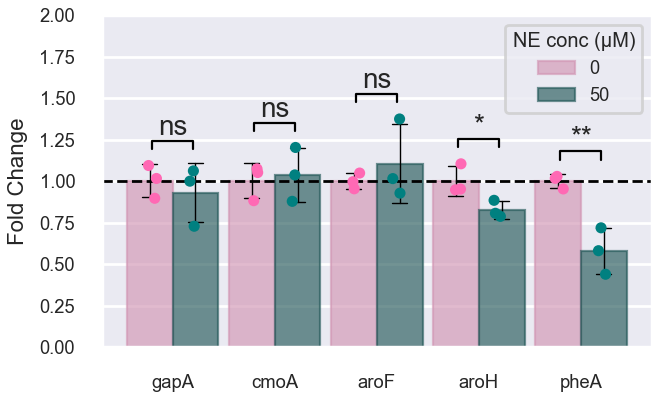


**Figure S5.** Extracted ion chromatograms obtained from LC-MS/MS analysis of carboxy-SAM in (A, B) synthetic standard, (C, D) the cell lysates of EHEC EDL933 cultured in medium without NE, and (E, F) the cell lysates of EHEC EDL933 cultured in medium supplemented with 50 µM NE.

| **Peak Area** | |
| --- | --- |
| **control** | **50 NE** |
| 5794.455 | 6426.725 |
| 5922.075 | 3188.81 |
| 4813.65 | 6942.7 |
| 7838.075 | 2241.395 |
| 4720.625 | 1915.445 |
| 7615.705 | 3197.19 |


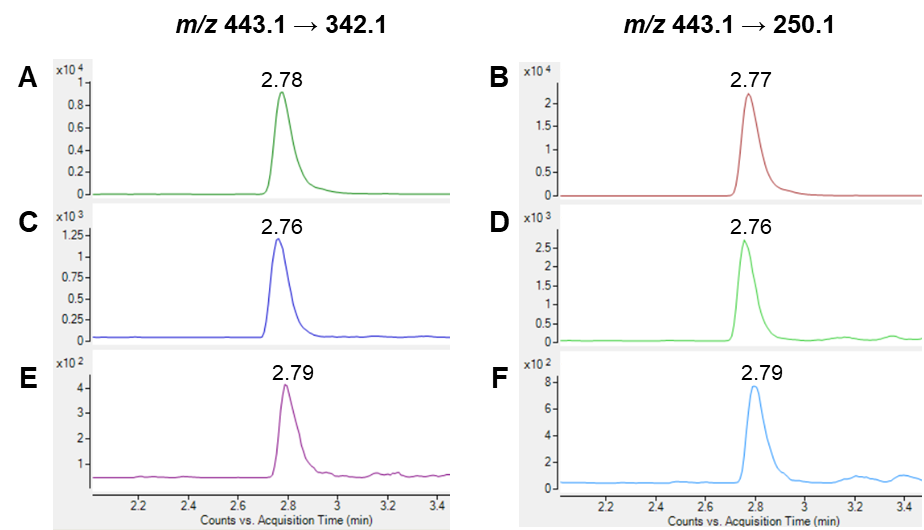


**Figure S6.** MS/MS spectrum obtained from collision-induced dissociation of the ion at *m/z* 443.1340. Shown alongside are the predicted fragmentation pattern of carboxy-SAM and a table comparing the observed and theoretical *m/z* values of the fragment ions.


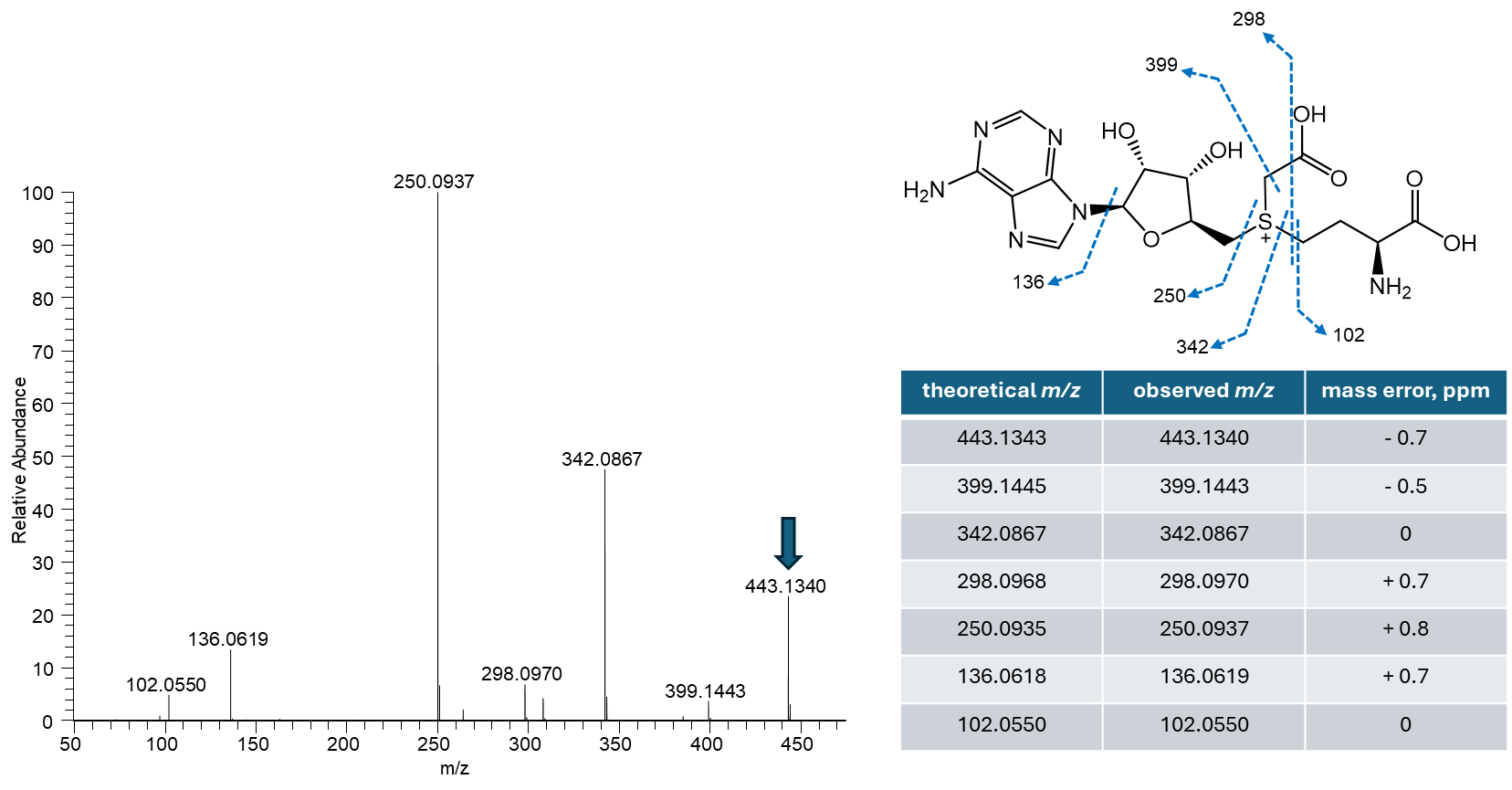


**Supplementary Table 1. Average codon usage frequencies (amino acid specific) of non-LEE vs. LEE genes.** Frequencies reflect the proportion of times the specified codon is used for each instance of its corresponding amino acid. Codons shown are read by tRNAs modified with (m)cmo^5^U, Q, or I as demonstrated in Figure 4. P-values from Student’s 2-sample, 2-tailed t-test.

|  | **Not LEE** | **LEE** | **pval** |
| --- | --- | --- | --- |
| **CCG** | **0.47** | **0.15** | **5.8E-12** |
| CCC | 0.14 | 0.11 | 4.1E-01 |
| **CCA** | **0.20** | **0.34** | **1.1E-05** |
| **CCU** | **0.18** | **0.36** | **3.8E-09** |
| **GCG** | **0.32** | **0.20** | **1.4E-05** |
| **GCC** | **0.26** | **0.19** | **3.6E-03** |
| **GCA** | **0.23** | **0.33** | **9.6E-05** |
| **GCU** | **0.18** | **0.28** | **4.1E-05** |
| ACG | 0.27 | 0.21 | 7.0E-02 |
| **ACC** | **0.39** | **0.13** | **8.2E-13** |
| **ACA** | **0.16** | **0.44** | **4.1E-21** |
| ACU | 0.18 | 0.23 | 8.1E-02 |
| **GUG** | **0.36** | **0.20** | **1.3E-06** |
| **GUC** | **0.21** | **0.16** | **3.1E-02** |
| **GUA** | **0.16** | **0.22** | **6.7E-03** |
| **GUU** | **0.27** | **0.42** | **8.3E-08** |
| UCG | 0.14 | 0.12 | 4.4E-01 |
| **UCC** | **0.15** | **0.06** | **8.9E-05** |
| **UCA** | **0.14** | **0.20** | **8.8E-03** |
| **UCU** | **0.15** | **0.26** | **2.1E-06** |
| **CUG** | **0.48** | **0.17** | **4.3E-17** |
| CUC | 0.10 | 0.09 | 5.2E-01 |
| **CUA** | **0.04** | **0.09** | **5.7E-06** |
| CUU | 0.12 | 0.15 | 9.3E-02 |
| **AAC** | **0.51** | **0.31** | **1.3E-05** |
| **AAU** | **0.48** | **0.69** | **3.0E-06** |
| **GAC** | **0.36** | **0.19** | **1.1E-06** |
| **GAU** | **0.63** | **0.81** | **4.7E-07** |
| **CAC** | **0.39** | **0.22** | **8.8E-04** |
| CAU | 0.55 | 0.62 | 2.0E-01 |
| **UAC** | **0.40** | **0.23** | **2.5E-04** |
| **UAU** | **0.56** | **0.74** | **2.2E-04** |
| **CGG** | **0.11** | **0.04** | **3.2E-03** |
| **CGC** | **0.35** | **0.15** | **2.6E-08** |
| **CGA** | **0.08** | **0.12** | **2.2E-02** |
| **CGU** | **0.35** | **0.26** | **1.4E-02** |

**Supplementary Table 2. Buffer gradient used in the LC-MS/MS of ribonucleosides.**

| **Time (min)** | **%A** | **%B** | **Flow rate (mL/min)** |
| --- | --- | --- | --- |
| 0 | 100 | 0 | 0.300 |
| 5 | 99 | 1 | 0.300 |
| 6 | 98 | 2 | 0.300 |
| 7 | 97 | 3 | 0.300 |
| 8 | 95 | 5 | 0.300 |
| 9 | 93 | 7 | 0.300 |
| 10 | 90 | 10 | 0.300 |
| 12 | 88 | 12 | 0.300 |
| 13 | 85 | 15 | 0.300 |
| 15 | 80 | 20 | 0.300 |
| 16 | 25 | 75 | 0.300 |
| 17 | 0 | 100 | 0.300 |
| 18 | 0 | 100 | 0.300 |
| 20 | 0 | 100 | 0.300 |
| 21 | 100 | 0 | 0.300 |
| 25 | 100 | 0 | 0.300 |

**Supplementary Table 3. Parameters for LC-MS/MS analysis of nucleosides.**

| **Compound** | **Precursor** | **Product** | **RT (min)** | **Compound** | **Precursor** | **Product** | **RT (min)** |
| --- | --- | --- | --- | --- | --- | --- | --- |
| 15N-dA | 257 | 141 | 4.6 | m5C | 258 | 126 | 1.46 |
| ac4C | 286 | 154 | 7.87 | m5s2U | 275 | 143 | 8.21 |
| acp3U | 346 | 214 | 1.37 | m5U | 259 | 127 | 4.16 |
| Am | 282 | 136 | 7.47 | m66A | 296 | 164 | 11.4 |
| Cm | 258 | 112 | 2.84 | m6A | 282 | 150 | 8.91 |
| cmnm5s2U | 348 | 141 | 2.39 | m6t6A | 427 | 295 | 10 |
| cmnm5U | 332 | 200 | 0.98 | m7G | 298 | 166 | 2.38 |
| cmo5U | 319 | 187 | 3.25 | mcmo5U | 333 | 201 | 9.3 |
| D_115 | 247 | 115 | 0.88 | mnm5s2U | 304 | 172 | 1.76 |
| Gm | 298 | 152 | 7.19 | mnm5U | 288.1 | 156.1 | 0.833 |
| ho5U | 261 | 129 | 1.18 | mo5U | 275 | 143 | 4.56 |
| I | 269 | 137 | 3.92 | ms2i6a | 382.2 | 250.1 | 17.1 |
| i6A | 336 | 204 | 16.5 | nm5s2U | 290 | 158 | 1.29 |
| io6A | 352.1 | 220 | 12.3 | preQ1 | 312 | 163 | 2.2 |
| m1A | 282 | 150 | 1.42 | Q | 410 | 163 | 5.1 |
| m1G | 298 | 166 | 7.24 | s2C | 260 | 128 | 1.93 |
| m22G | 312 | 180 | 9.71 | s2U | 261 | 129 | 4.42 |
| m2A | 282 | 150 | 6.42 | s4U | 261 | 129 | 5.033 |
| m2G | 298 | 166 | 8.0 | t6A | 413 | 281 | 12.5 |
| m3C | 258 | 126 | 1.23 | Um | 259 | 113 | 5.7 |
| m3U | 259 | 127 | 5.8 | Y | 245 | 291 | 0.9 |

**Supplementary Table 4. Peptide fractionation gradient.** A total of 80 fractions were collected every minute starting at minute 10.

| **Time (min)** | **% B** | **Flow rate (μL/min)** |
| --- | --- | --- |
| 0 | 1 | 200 |
| 2 | 1 | 200 |
| 10 | 5 | 200 |
| 72 | 35 | 200 |
| 87 | 70 | 200 |
| 92 | 70 | 200 |
| 93 | 1 | 200 |
| 100 | 1 | 200 |

**Supplementary Table 5. Fractionated peptide pooling scheme.** 80 total collected fractions were consolidated into 8 fractions for analysis.

| Fraction 1 | 1 9 17 25 33 41 49 57 65 73 |
| --- | --- |
| Fraction 2 | 2 10 18 26 34 42 50 58 66 74 |
| Fraction 3 | 3 11 19 27 35 43 51 59 67 75 |
| Fraction 4 | 4 12 20 28 36 44 52 60 68 76 |
| Fraction 5 | 5 13 21 29 37 45 53 61 69 77 |
| Fraction 6 | 6 14 22 30 38 46 54 62 70 78 |
| Fraction 7 | 7 15 23 31 39 47 55 63 71 79 |
| Fraction 8 | 8 16 24 32 40 48 56 64 72 80 |

**Supplementary Table 6. Proteomics nLC gradient.** Sample pickup volume was 1 microliter at a flow of 10 microliter/min. Sample loading volume was 4 microliters at a max pressure of 500 bar. Precolumn equilibration volume was 10 microliters, and analytical column equilibration volume was 4 microliters.

| **Time (min)** | **Duration** | **Flow (nL/min)** | **%B** |
| --- | --- | --- | --- |
| 0 | 0 | 400 | 2 |
| 5 | 5 | 400 | 5 |
| 105 | 100 | 400 | 25 |
| 125 | 20 | 400 | 37 |
| 129 | 4 | 400 | 50 |
| 130 | 1 | 400 | 95 |
| 146 | 16 | 400 | 95 |

**Supplementary Method 1. Chemical synthesis and characterization of carboxy-SAM**

Carboxy-SAM was synthesized as previously reported by Kim *et al.* with modifications.^1^ Briefly, *S*-adenosyl-L-homocysteine (6 mg) was dissolved in 1 mL of 150 mM ammonium bicarbonate before the addition of iodoacetic acid (200 mg). The reaction mixture was incubated with gentle rocking at 37 ℃ for 24 h and subsequently purified using a combination of hydrophilic interaction and reversed‐phase liquid chromatography. The purified fractions were combined, dried with a SpeedVac concentrator, and reconstituted in 10 mM ammonium formate (pH 3.6). The reaction product was analyzed by high-resolution MS, revealing excellent agreement between the observed *m/z* (443.1340) and the theoretical *m/z* (443.1343) for the protonated molecular ion of carboxy-SAM, with a mass error of less than 1 ppm. MS/MS analysis of the ion *m/z* 443.1340 also yielded a fragmentation pattern consistent with the predicted fragments of carboxy-SAM as depicted in Figure S5, confirming the reaction product as carboxy-SAM.

**Supplementary Method 2. LC-MS/MS analysis of carboxy-SAM**

The analysis was performed on an Agilent 1290 HPLC system coupled to an Agilent 6495 triple quadrupole mass spectrometer. Prior to the analysis, the dried sample residues were reconstituted in 30 µL of 10 mM ammonium formate (pH 3.6), vortex-mixed, and centrifuged at 12,000 *g* for 5 min. Ten microliters of each sample were injected onto a Phenomenex Luna Omega 1.6 µm Polar C18 column (2.1x150 mm i.d.) operated at 35 ℃ with a flow rate of 250 µL/min, using a gradient elution of acetonitrile in 10 mM ammonium formate (pH 3.6) as follows: 0–4 min, 0%; 4–10 min, 0–100%; 10-14 min, 100%, followed by an 8-min re-equilibration to the initial condition. The JetStream ESI source was operated in positive-ion mode with the following optimized parameters: drying gas temperature, 180 °C; gas flow, 15 L/min; nebulizer, 40 psi; sheath gas temperature, 350 °C; sheath gas flow, 12 L/min; capillary voltage, 2000 V; nozzle voltage, 2000 V; and HP/LP iFunnel RF, 190/70 Vp-p. The MS was operated in MRM mode with the following transitions for carboxy-SAM: *m/z* 443.1 → 342.1 (quantitative; collision energy, 7 V) and *m/z* 443.1 → 250.1 (qualitative; collision energy, 10 V). Results from two technical replicate injections were averaged.

**Supplementary Method 3. RT-qPCR of shikimate pathway associated genes.**

Overnight EHEC EDL933 cultures were diluted 1:100 and grown in DMEM low glucose, 37C, shaking at 180rpm in 0 and 50 µM NE. RNA was extracted from 500 microliters of culture using 1 mL of Trizol. Total RNA was purified from the aqueous phase using the Monarch Spin RNA cleanup kit (NEB T2030L), following kit instructions. Contaminating gDNA was removed using the TURBO DNA-free kit (ThermoFisher AM1907). Approximately 300 ng of input RNA was used to generate cDNA using the ProtoScript First Strand cDNA synthesis kit (NEB E6300S), following kit instructions.

Approximately 10 ng of cDNA was added to a 10 μL reaction containing 300 nM of forward and reverse primer and PowerUp SYBR Green Mastermix (ThermoFisher A25741). RT-qPCR reactions were performed in technical triplicate in 384-well plate format on a Lightcycler 480 system (Roche). Fold-change relative to the gyrA housekeeping gene was calculated using the ddCT method.

The following primer pairs for each gene were used:

gyrA:

F: GAAAACCGTCCTCACCGAGT

R: ATTACTTCGTCTTCGCCGCT

gapA:

F: GTTGACCTGACCGTTCGTCT

R: ACGTCATCTTCGGTGTAGCC

cmoA:

F: CGGTTACAGCGAACTGGAGA

R: GGCGTGCTTTATGGGTTTCC

aroF:

F: ATCAGTCTTCCGAGCAACCG

R: GCAAGGCATCGGTCATTTCC

aroH:

F: CTCTACGGCGATTGCTGGAA

R: GACCAGGCGTTCGGTATCTT

pheA:

F: AAATCACCGCATGTTGCTGC

R: TCGGGTGAAGTTTTGTCGCT

**Supplementary References**

1. Kim, J. *et al.* Determinants of the CmoB carboxymethyl transferase utilized for selective tRNA wobble modification. *Nucleic Acids Res* **43**, 4602–4613 (2015).
